# Developing algorithmic psychiatry via multi-level spanning computational models

**DOI:** 10.1016/j.xcrm.2025.102094

**Published:** 2025-04-28

**Authors:** Michael M. Halassa, Michael J. Frank, Philippa Garety, Dost Ongur, Raag D. Airan, Gerard Sanacora, Kafui Dzirasa, Sahil Suresh, Susan M. Fitzpatrick, Douglas L. Rothman

**Affiliations:** 1 Department of Neuroscience, Tufts University School of Medicine, Boston, MA, USA; 2 Department of Psychiatry, Tufts University School of Medicine, Boston, MA, USA; 3 Department of Cognitive and Psychological Sciences, Carney Institute for Brain Sciences, Brown University, Providence, RI, USA; 4 Institute of Psychiatry, Psychology and Neuroscience, King’s College London, London, UK; 5 McLean Hospital and Harvard Medical School, Boston, MA, USA; 6 Department of Radiology, Stanford University School of Medicine, Stanford, CA, USA; 7 Department of Psychiatry and Behavioral Sciences, Stanford University School of Medicine, Stanford, CA, USA; 8 Department of Psychiatry, Yale University School of Medicine, New Haven, CT, USA; 9 Department of Neurobiology, Duke University Medical Center, Durham, NC, USA; 10 Department of Psychiatry and Behavioral Sciences, Duke University Medical Center, Durham, NC, USA; 11 James S. McDonnell Foundation, St. Louis, MO, USA; 12 Department of Biomedical Engineering, Radiology and Biomedical Imaging, Yale University, New Haven, CT, USA

## Abstract

Modern psychiatry faces challenges in translating neurobiological insights into treatments for severe illnesses. The mid-20th century witnessed the rise of molecular mechanisms as pathophysiological and treatment models, with recent holistic proposals keeping this focus unaltered. In this perspective, we explore how psychiatry can utilize systems neuroscience to develop a vertically integrated understanding of brain function to inform treatment. Using schizophrenia as a case study, we discuss scale-related challenges faced by researchers studying molecules, circuits, networks, and cognition and clinicians operating within existing frameworks. We emphasize computation as a bridging language, with algorithmic models like hierarchical predictive processing offering explanatory potential for targeted interventions. Developing such models will not only facilitate new interventions but also optimize combining existing treatments by predicting their multi-level effects. We conclude with the prognosis that the future is bright, but that continued investment in research closely driven by clinical realities will be critical.

## Graphical abstract

**Figure:**
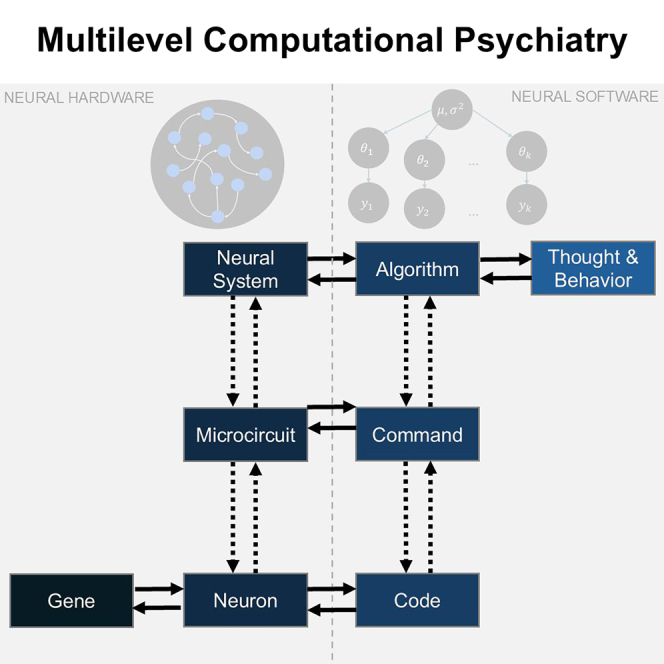

## Teaser

Halassa et al. introduce algorithmic psychiatry as a framework for mental illness. Using schizophrenia as a case study, they demonstrate how predictive processing integrates neural and cognitive data to drive targeted interventions. This approach could revolutionize psychiatric care, offering new hope for personalized treatments and improved outcomes.

## Introduction

After his monthly paliperidone injection, Robert goes back to his room and lies down for the rest of the day. He seldom interacts with others and spends much of his time pacing the hallway. Though he now engages with staff and is less internally preoccupied, he cannot leave the facility he resides in. Before hospitalization, Robert’s disruptive behavior strained his family relationships, and they will not take him back. While medications have lessened his psychotic symptoms, they have not restored his functional capacity, leaving him unemployed for years. Like many with schizophrenia, he lives a life of uncertainty, unsure he will ever gain independence, hold down a job, or build meaningful relationships. Robert is all too representative of the many individuals who, despite great effort by researchers and clinicians, remain debilitated by this profound disorder.

As a medical discipline, psychiatry can claim some progress over the past century, emerging from the eras of asylum care into an era marked by the development of several classes of psychoactive pharmaceuticals.^1^^,^^2^ Major psychiatric disorders, such as mood, anxiety, and psychotic disorders, have benefited from drug development, with many overcoming or reducing the frequency of symptomatic episodes that, untreated, would have severely compromised their ability to fully participate in society. More recently, evidence-based psychotherapies and interventions like transcranial magnetic stimulation (TMS) and deep brain stimulation have further improved therapeutic outcomes.^3^^,^^4^^,^^5^ These new technologies have been complemented by recent advances in precision neurophysiology and neuroimaging^6^^,^^7^ for mental health characterization, and precision noninvasive intervention and functional mapping of the brain.^8^

Despite these successes, schizophrenia remains a large unmet public health need. Schizophrenia is a condition that attacks the very notion of what it is to be a person with agency over our senses and actions; patients often suffer from hallucinations (commonly auditory) and delusions (persistent false beliefs despite contrary evidence). These features, referred to as positive symptoms, co-exist with a host of negative ones in which patients appear apathetic and withdrawn but without a clear affective expression or anxiety. Although antipsychotic medications do provide some symptom relief (albeit less so for negative ones), almost all patients continue to exhibit cognitive deficits that prohibit their independence. In about 30% of patients, even psychotic symptoms cannot be adequately controlled, leading to “treatment resistance,” which can condemn people to homelessness, state hospital residency, or total reliance on family care. Recently, the Food and Drug Administration approved xanomeline/trospium (Cobenfy), the first non-dopaminergic treatment targeting muscarinic pathways, showing promise for negative and cognitive symptoms.^9^ While exciting, it will take several years before we’re able to evaluate the impact of this advance. Critically, availability of new medications does not fundamentally alter the arguments we present in this article.

In contrast to the slow clinical progress, brain research has advanced rapidly in recent decades, driven by technologies enabling the interrogation of the brain and mind from novel perspectives.^10^ The rise of approaches in animals for observing and perturbing the activity of synapses, neurons, and populations within intact circuits and networks has been nothing short of a scientific revolution.^11^ Guided by human genomics, these studies have identified promising pharmaceutical targets.^12^^,^^13^ In addition, neuroimaging methods such as positron emission tomography (PET), electroencephalography (EEG), and magnetic resonance imaging/spectroscopy (MRI/MRS) now allow direct visualization of alterations in the brain chemistry and function in human patients, as well as the impact of new treatments.

Why hasn’t this tremendous progress in research translated to similar advances in patient care? Following the discovery of the first effective antipsychotic medication chlorpromazine, hope was placed on the notion that medications could target specific molecular alterations and correct existing brain chemical imbalances. As research identified abnormalities in dopamine (DA), glutamate, and other neurochemical systems in schizophrenia, drug development focused on these targets. Unfortunately, this approach has not produced genuine breakthroughs, prompting many to declare a crisis in schizophrenia treatment development.^14^^,^^15^

**Figure 1:**
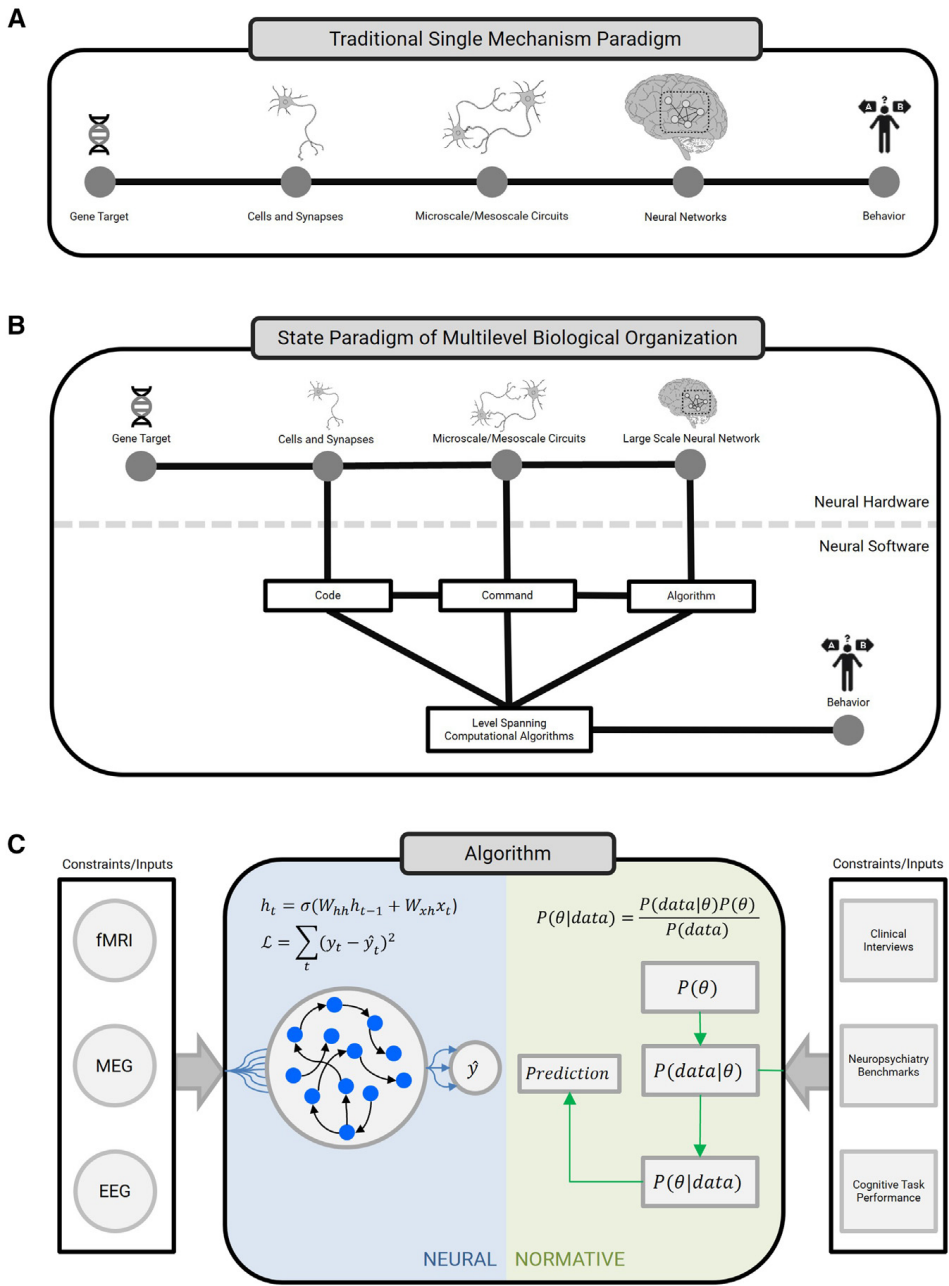
An algorithmic framework for connecting neurobiological substrates to thought and behavior (A) Traditional paradigm, where genes function within cells, giving rise to circuits that operate within networks, which collectively give rise to behavior. This paradigm emphasizes that genetic lesions can be targeted by (pharmacological) approaches for treatment and implicitly assumes alterations occurring in higher levels will normalize. (B) State paradigm for multi-level biological organization, emphasizing the parallel nature of computations across levels. The distinction between neural hardware (biological structures) and neural software (functional computations) is illustrated to further illustrate models that span across these levels. (C) Focus on the algorithmic level, which is closest to observable behavior and offers the most immediate opportunities for altering disease trajectories. Behavior-relevant algorithms can come in a neural form, which involve task-optimized models solving various behavioral tasks and provide comparable data to human subject (or animal) data. Normative models of behavior provide a complementary perspective; they are non-neural and their Bayesian machinery can provide interpretable quantities, which also provide insight into the strategies taken by subjects. We suggest that the integration of these two types of models will be critical for the future of behavioral description in psychiatry, as well as their initial mapping onto neural substrates.

Given these findings, critics increasingly argue that schizophrenia research has become overly reductionist, unable to address the heterogeneous, dynamic nature of mental illness.^16^^,^^17^ Such criticism is often directed at a traditional molecular-focused paradigm in which a genomic lesion propagates across multiple levels of organization to impact behavior (Figure 1A). Critics challenge the assumption that higher level abnormalities can be corrected by single molecular interventions at lower levels, emphasizing that many aspects of mental illness, especially interactions between individuals and their social environment, are beyond reductionist approaches. In contrast, advocates of the molecular (now extended to -omics) paradigm have argued that the problem is not too much emphasis on reductionist science but instead not enough, criticizing the alternate “holistic” view as being too vague to lead to useful treatments.^18^^,^^19^ In this paper, we advocate a perspective centered around algorithmic, “latent variable” models of behavior. However, unlike pure behavioral state models, we advocate integrating the behavioral description with advances in computational modeling at the level of cells and circuits to improve our ability to predict the impact of molecular and regional targeted treatments (Figure 1B).

Such models assume a stepwise nature of human cognition as a sequence of unobservable latent states that can be decomposed into a list of computational functions (short term memory, integration, feedback, etc. …). These models have been developed to describe behavior in various types of tasks, providing advantages over more qualitative descriptors.^20^^,^^21^^,^^22^ First, they provide ways to group task states via a common computation instead of simply averaging unrelated states.^23^ Second, such state grouping provides a way for correlation with neural measurements, linking brain and behavior through computational language.^24^^,^^25^^,^^26^ Third, psychiatry already implicitly uses latent descriptors to guide treatment. For example, when evaluating depressed mood, the astute psychiatrist avoids symptom-triggered treatment using antidepressants and instead clarifies whether this is part of a bipolar syndrome that would require mood stabilizers instead. Psychiatric diagnostic entities address multiple symptoms with a single medication, reducing polypharmacy. Therefore, the adoption of algorithmic models would naturally align with current treatment optimization practices.

Algorithmic behavioral models are at the center of multi-level spanning computational models, which can incorporate data across the levels of organization relevant to psychiatry. The time is ripe for such developments, given the revolution in computational power and deep learning approaches.^27^ By integrating all the traditional levels of organization in this framework, psychiatry can potentially achieve what it heretofore has lacked—a vertically integrated theory of brain function that links molecular structures with neural functions, neural functions with cognitive operations, and cognitive operations with real-world behavior (Figures 1B and 1C). Consider an active working memory task in which subjects must remember and update a list of items. An algorithmic model may break down this task into latent cognitive states such as maintenance, updating, and response selection. While these latent states are not directly observable, behavioral data allow us to infer them. Below this algorithmic level, we have the command level, which specifies the computational operations that implement these latent states, such as neural integration,^28^ line,^29^ or bump attractors.^30^ These computational operations can be directly tested against empirical neural data and further linked to single-cell neural encoding properties at the level beneath. Critically, out of all these levels, the algorithmic level is the one that is closest to behavior, and therefore, it can have clear inroads to behavioral data fitting on one end and neural modeling on another (Figure 1C).

Because biological data are described in a manner that is not intended to inform psychological function, translating biological knowledge into computational terms is essential to this framework. In fact, the parallel revolution in computer science and artificial intelligence not only provides tools to examine how the underlying neurobiology works but also the appropriate language to link biology with psychology.^31^^,^^32^ While the higher level component descriptions of behavior derive from cognitive psychology and therefore are intrinsically describable in the language of stepwise computational processes, advanced neuroimaging studies have not found equivalent neuroanatomical representations.^33^ Therefore, even if an impaired computational process is identified by behavioral studies, the anatomical location and biological level for optimal intervention are no longer clear with the loss of a 1:1 function to anatomical location relationship. However, imaging studies have linked behaviors to distributed anatomical networks of neuronal activity. The intrinsically parallel multi-level algorithms of modern deep learning approaches are potentially ideal and likely essential for relating networks to latent variable models of behavior as well as to lower circuit and synapse levels of description (Figure 1C). Additionally, leveraging rapid advances in anatomically targeted therapies, as discussed in Conclusions and future outlook, is crucial.

In the rest of this piece, we first provide an overview for the role of formal computational theory in describing cognitive operations that link to behavior. Next, we illustrate how this approach can augment an ongoing success story that takes inspiration from behavioral economics to target abnormal thinking in schizophrenia. We then move to progressively lower levels of mechanistic description, from large-scale brain networks measured by methods such as fMRI to molecular mechanisms at the level of receptors and genes to describe how new experimental approaches and statistical tools are revolutionizing these areas and building bridges between them. We end with speculations on how all these advances will inform treatment.

## Present-level spanning computational approaches and their application to treatment

In this section, we summarize several approaches using computational models to bridge across levels of analysis. The discussion is not meant to be comprehensive, but rather illustrates examples of key achievements and current limitations experienced to date.

### The role of formal computational theory in schizophrenia, linking cognitive theory to behavior

While much progress has been made in identifying biological alterations in the pathophysiology of schizophrenia, such findings alone do not explain how they translate into functional outcomes or symptoms that characterize schizophrenia and mental illness more broadly. The complexity of systems neuroscience implies that even neurological diseases with “high essentiality” (i.e., well-defined etiology traced to a single or small number of mechanisms^34^^,^^35^) can have complex interactive effects on cognition and behavior. A canonical example is Parkinson’s disease (PD), which results primarily from degeneration within midbrain DA neurons, but which percolates throughout corticostriatal circuits and thus leads to alterations across motor, affective, and cognitive functions.^36^ While primarily characterized by a set of stereotypical motor symptoms, the cognitive, affective, and perceptual consequences of PD are heterogeneous.^37^^,^^38^ This poses a treatment challenge not unlike that seen in schizophrenia, particularly that traditional antipsychotic treatments need to be balanced against worsening motor symptoms. Thus, even a known lesion, such as the degeneration of DA neurons in an identified neuroanatomical location, can yield heterogeneous symptoms whose circuit-level explanation remains an open question and where treatment is just as complex as that of less molecularly understood disorders such as schizophrenia.

Formal computational models can help address the problem of heterogeneity and unify seemingly disparate symptom clusters in PD. They can do so by reducing the complexity of the circuits to their functional considerations shared across such circuits, with analogous effects of DA modulation therein.^39^ These models, spanning detailed neural circuits to algorithmic descriptions of action selection,^40^ have inspired studies to test their predicted implication of valuation and reinforcement learning in the progression of Parkinsonian symptoms. Such approaches have been applied to both patients with PD and to patients experiencing Parkinsonian symptoms because of antipsychotic treatment in schizophrenia. Predictions of these models have been confirmed in rodent models. For example, the models correctly predicted that motor dysfunction would advance as a function of aberrant learning (exaggerated plasticity) in striatal D2 receptor-expressing spiny neurons and that such deficits can be separated from the direct effects of DA denervation on “performance.”^41^^,^^42^ Similarly, they have accounted for analogous effects on cognitive function and decision-making via multiple corticostriatal circuits^43^ and the paradoxical finding that DA medications can have both therapeutic and deleterious effects depending on task demands.^40^^,^^44^

**Figure 2:**
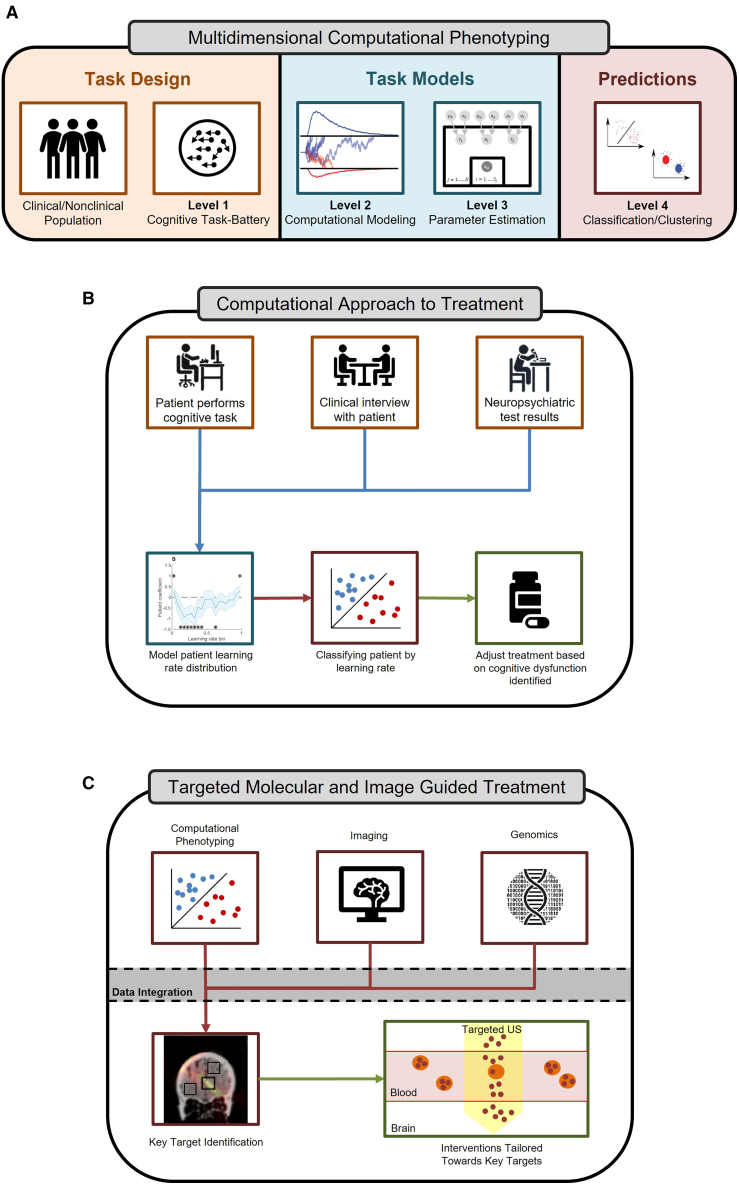
A framework for a computational approach to treatment guidance (A) Multidimensional computational phenotyping. Depiction of computational phenotyping beginning with task design where clinical and nonclinical populations undergo task batteries designed to probe specific cognitive functions and capture behavioral data. The data are subjected to modeling to simulate cognitive processes and identify key parameters in performance and output. The parameters are parsed to quantify individual differences in cognitive processes, and the individuals are classified and clustered based on the models to allow for personalized predictions. (B) Computational approach to treatment. Illustration of how phenotyping can guide treatment decisions. Cognitive task performance from patients is complemented with additional clinical data for modeling, which allows for cognitive behavioral classification. Treatment is adjusted according to the dysfunctions identified in a personalized manner. (C) Targeted molecular and image-guided treatment. By combining computational phenotyping with advanced functional imaging and genomics, clinicians can design integrated targeted therapies (e.g., using localized ultrasound for targeted drug release).

This same strategy to compress multiple interacting cellular and systems-level circuits into latent low-dimensional models that summarize their core computations (“multidimensional computational phenotyping”) has improved prediction and diagnostics in more complex mental illness,^45^^,^^46^ including schizophrenia^47^ and depression^48^ (Figure 2). However, it becomes increasingly difficult to link such functional considerations to a small number of reductionist mechanisms. In the language of Hitchcock et al., schizophrenia represents an illness of low to moderate “essentiality”: there are some consistent findings on the pathophysiology, but it certainly cannot be reduced to a single set of mechanisms. The essence of the illness is more likely to be found in the heterogeneity across patients.^34^ Thus, successful predictive models have largely taken the “quantitative abductive” approach,^22^^,^^49^^,^^50^ beginning with algorithmic models of behavior, fitting them to behavioral data, and determining post hoc which model parameters best discriminate between patient populations, without drawing strong conclusions about mechanism.

Nevertheless, sometimes observed parameter differences can plausibly be linked to a neural circuit or set of mechanisms, either because neural recordings were used in the associated study and their variations were linked to model parameters,^48^^,^^51^ or because the observed parameter differences have well-established links to underlying mechanisms. As a disorder of moderate essentiality, schizophrenia has long been linked to disturbances of the prefrontal cortex (PFC)^52^ as well as the DA system.^53^^,^^54^ Formal models of corticostriatal DA systems have been extended to provide an account of the DA hypothesis of schizophrenia, considering how multiple aspects of positive and negative symptoms may arise from disruptions to DA within individual circuits and on different time scales.^55^ But here one should think of this approach as “how far can one get” with a simple model rather than a comprehensive account of schizophrenia. Indeed, task paradigms, combined with formal models designed to disentangle striatal DA mechanisms from prefrontal cortical contributions, have consistently failed to show clear alterations of DA-dependent reinforcement learning processes in (chronic) schizophrenia and instead attribute alterations in learning deficits in prefrontal cortical working memory processes.^56^^,^^57^ Given the extensive interactions among these systems and with hippocampal and other circuits, drawing precise causal conclusions from any single task or patient group remains challenging.

One might argue that such studies often involve chronically medicated patients, whose intact striatal DA-dependent processes could simply reflect normalization by antipsychotics (D2 receptor antagonists preferential targeting in the striatum rather than the PFC). But despite decades of research and clinical trials, next-generation antipsychotics have seen limited progress. This may be understood from the perspective of next-generation computational models and systems neuroscience approaches highlighting that a single blunt manipulation of DA or other modulators is unlikely to completely ameliorate symptomology.

Consider that within the DA system, traditional reinforcement learning models and data relied on the notion that DA signals conveying salient “reward prediction error” signals^58^^,^^59^ are *scalar* (reflecting a single number that goes up or down) and *global* (influencing all downstream targets across the striatum and elsewhere equally). Recent studies in animal models have challenged this notion however, with endogenous DA signals showing substantial heterogeneity, in part depending on their downstream targets.^60^^,^^61^^,^^62^^,^^63^ Moreover, even within localized regions of the dorsal striatum, rewards induce wave-like spatiotemporal dynamics of DA, with the direction of the waves (from medial to lateral or vice versa) dependent on task demands.^64^ When the task requires *agency*—the animal controls how they progress from one state to another before receiving rewards—DA waves initiate within the dorsomedial striatum (DMS). But when task events indicating progress to reward were incongruent with the animals’ actions, the DMS was the last region to receive DA. These opponent wave dynamics were complemented by more gradual DA *ramps* in the DMS that led up to the reward as the animal progressed in the task, again in opponent directions (increasing ramps in the agency condition, decreasing ramps in the passive condition). Finally, brief DA transients in individual axon segments localized to DMS subregions as animals experienced individual events signaling discrete progress toward reward.^64^^,^^65^ A “mixture of experts” computational model synthesized how such transients, ramps, and waves interacted in the service of directing “credit assignment” to the appropriate striatal subregion, allowing the animal to learn whether they are in control of task events and how to guide and reinforce behavior accordingly. Model predictions, in terms of how the different DA dynamics were predictive of each other and of subsequent behavioral adjustment, were supported by observed experimental data.

### One level up: Taking inspiration from behavioral economics to target abnormal thinking in schizophrenia

What does the aforementioned computational approach mean to Robert, who, like many others, struggles to live to his full potential as a consequence of his condition? We seek answers in biological alterations, but as noted earlier, such findings alone are insufficient to explain how they translate into symptomatic or functional outcomes. Is it useful to ask how his symptom of inactivity relates to his conscious thoughts and feelings, if at all? Suppose Robert reports that he does not go out because he is fearful, believing that he is being followed by people with malicious intent, and the reason for this is that he often hears isolated unidentifiable voices, telling him to “go home.” Staying home feels safer, despite the negative repercussions, and over the years, it has become habitual, further limiting his social functioning. The computational approach means that for individuals like Robert, we can quantitatively identify and understand the ways in which his brain processes information abnormally.

Cognitive processes and phenomenological experiences form a crucial component of vertically integrated models, linking biological vulnerability, social factors, and the manifestation of individual psychotic symptoms.^66^ Over the past two decades, a number of converging cognitive models of psychosis^66^^,^^67^^,^^68^^,^^69^ propose that beliefs and appraisals are central to determining the clinical consequence of psychotic experiences. These cognitive models of psychosis recognize biological alterations as conferring an increased vulnerability to disordered perceptions (e.g., hearing a disembodied voice). However, disordered perceptions alone are not psychotic (for example, benign voices are a relatively common human experience).^70^ How individuals make sense of, and respond to, anomalous experiences that characterize psychosis can determine whether they remain benign or result in impairment. The conclusions that Robert believes the voices he hears derive from individuals who intend harm and follow him about are typically considered delusions. These are distressing and lead him to adhere to dysfunctional behaviors such as asociality. These appraisals, as such models suggest, stem from past experiences (e.g., of childhood trauma or later adversity) influencing content of appraisals (e.g., voices as malign). They are also influenced by biased reasoning, which may be due to cognitive limitations (e.g., working memory).

Reasoning processes have long been considered disrupted in schizophrenia (and were integral to the Diagnostic and Statistical Manual definition of delusions as “false beliefs *due to incorrect inference* about external reality” DSM-III and -IV). Systematic reviews and meta-analyses have detailed a large and consistent evidence base, in over 70 studies, in which the clear majority show that individuals with delusions and psychosis make decisions on the basis of limited evidence in probabilistic reasoning tasks, the so-called “jump-to-conclusions” (JTC) bias.^71^^,^^72^^,^^73^^,^^74^ This bias is posited to contribute to delusion formation; thus, it is proposed that anomalous or ambiguous information is rapidly appraised, and a delusional conclusion is drawn on the basis of limited evidence. Additionally, once a conclusion is drawn, even if implausible, it occurs without a thorough consideration of alternatives or a corrective review of the evidence, thus entrenching delusional persistence. This latter process of reviewing one’s initial impressions or beliefs has been termed “belief flexibility,” which research has also shown to be poor in people with delusions.^73^^,^^75^ We should note that there are more nuanced views on the JTC bias, highlighting basic differences in information seeking in patients with schizophrenia.^74^

This empirical research finds a clear parallel in the work of the behavioral economists Tversky and Kahneman, who in numerous studies have shown that people do not always make rational or optimal decisions even when the information is available to do so.^76^ They have documented many heuristics and biases in decision-making, which may lead people to irrational actions. Kahneman’s book, “Thinking Fast and Slow” popularized this work and distilled his body of research within the framework of two-process models of reasoning.^76^ In cognitive psychological theory, dual-process models of human reasoning posit two parallel systems or processes underpinning decision-making: type 1, fast, high capacity, independent of working memory and cognitive ability; and type 2, slow, low capacity, heavily dependent on working memory and related to individual differences in cognitive ability*.*^77^^,^^78^^,^^79^^,^^80^^,^^81^

If we consider reasoning in psychosis in this context, it is apparent that JTC may be considered to reflect the operation of type 1 *fast* processes, while the documented failure in people with delusions to correct mistaken judgments from conflicting evidence is a failure of type 2 *slow thinking* (i.e., the ability to consider alternative explanations and review the evidence thoroughly).^75^

**Figure 3:**
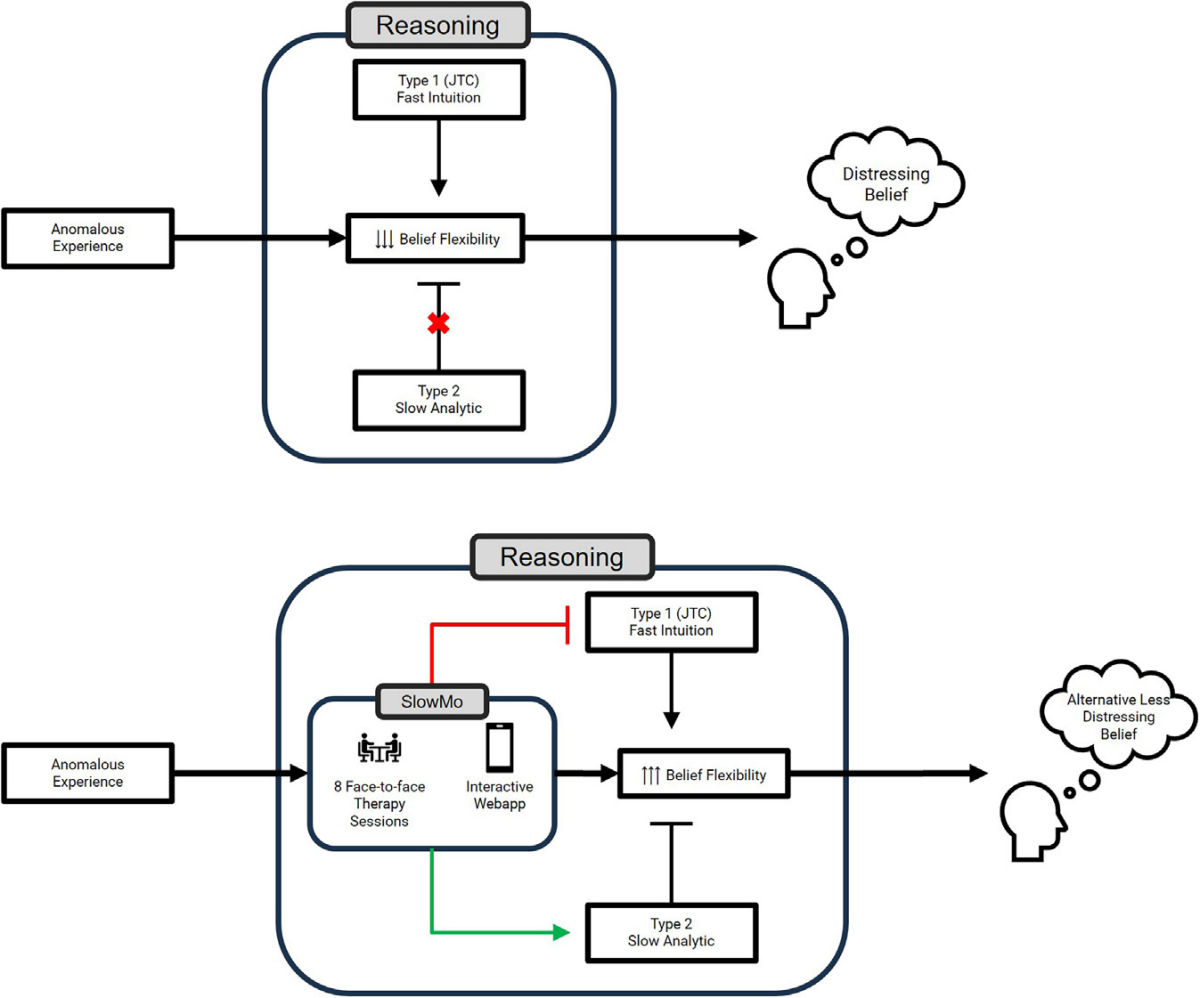
Dual-process model of reasoning Top: Fast and slow thinking in schizophrenia. A schematic representation of the dominance of fast intuition over slow analytic reasoning as it applies to poor belief flexibility and thus distressing beliefs about anomalous experiences. Bottom: Technology-assisted approach (SlowMo) for ameliorating reasoning dysfunction in schizophrenia. A schematic representation of the role of the dual-process treatment of SlowMo therapy. Through therapy sessions and a mobile application that acts as an “extended mind,” there is increased resource allocation toward slow, analytic thinking. This overrides the type 1 fast thinking and leads to alternative, less distressing beliefs, consequently mitigating cognitive dysfunction.

Stimulated by this research, a new digitally supported psychological intervention, “SlowMo,” was developed to help patients with schizophrenia manage delusions by targeting reasoning, specifically fast and slow thinking (Figure 3A). This is grounded in cognitive models of psychosis, and cognitive behavior therapy, but focuses intensively on the mechanisms of fast and slow thinking, bringing into a person’s awareness their tendency to jump to conclusions and providing tools for “slowing down” thinking and generating more benign alternative thoughts to the person’s current and distressing persecutory delusion (Figure 3B). SlowMo is a blended therapy with eight face-to-face therapy sessions and is supported by a web app with interactive materials, vignettes, and exercises. A linked mobile app encourages the user to enhance slow thinking in everyday life: providing ready access to a repository of personalized non-delusional safety ideas, previously generated in therapy, thus supporting effective self-management during distressing experiences. These interactive elements can be modified to specifically fit each patient’s reasoning style based on behavioral feedback. A clinical trial^82^ demonstrated significant improvements in persecutory delusions, overall functioning, and well-being and that one mechanism for this was through increased type 2 slow thinking, specifically belief flexibility.

The success of SlowMo supports that further work on integration of formal algorithmic modeling with higher cognitive theories that interface with real-world interventions would be of tremendous benefit. For example, computational phenotyping of patients with schizophrenia based on their task behavior, movement, speech patterns, and/or brain signals should help identify to what degree fast thinking style is exaggerated versus slow thinking impaired. By capturing the metrics of fast vs. slow thinking—such as decision thresholds, reconsideration times, and belief flexibility—the field can quantify the degree to which each reasoning style contributes to persistent delusional beliefs. These individualized metrics allow SlowMo to be tailored to target the specific cognitive processes most in need of intervention, thus resulting in the design of a personalized intervention, capable of iterative amelioration of psychotic experience. The dual-process model of reasoning can consequently be enriched by bridging these cognitive metrics with biological processes. Controlled paradigms of decision-making are essential to this integration, where subjects are presented with initially ambiguous images that are resolved over time. Under these conditions, patients with schizophrenia indeed jump to fast conclusions.^83^ However, developing paradigms in which participants may revise their conclusions could reveal additional deficits and formalize the notion of “belief reappraisal” biases in patients with schizophrenia. More broadly, creating paradigms with dissociable individual components facilitates algorithmic modeling of the dynamics of belief formation and revision. This accomplishes two goals: the individualized patient approach outlined previously and the link to the underlying brain mechanisms, as the fast thinking style may align better with BG mechanisms whereas the slow style with prefrontal ones.^84^ This has important implications for predicting treatment responses as well as the development of new interventions.

### Predictive processing theory as a bridge to mechanism

A promising set of models that have the capacity to link higher cognition to neural mechanism lies within the predictive processing framework.^85^ Several types of predictive processing theory (PPT) exist, but they share the core idea that perception lies at the interface between memory-based predictions and environmental sensory inputs.^86^ For example, responses in primary sensory cortex are thought to only partially reflect information from the periphery, as some are better explained by deviations from internal predictions.^87^ These findings can be attributed to the highly interconnected nature of cortical areas; higher-order sensory cortex that responds to more complex features generates more statistically probable stimuli based on partial inputs, which then interact with the lower level inputs in primary sensory areas. Deviations from these predictions are transmitted back to higher areas to update their activity (potentially requiring neuromodulators such as DA). Based on this iterative process, PPT has striking parallels to cognitive reappraisals discussed earlier. PPT makes predictions in this domain that have been verified by experiments, such as finding dedicated cortical neurons that respond to quantities that are higher or lower than memory-based predictions.^87^

**Figure 4:**
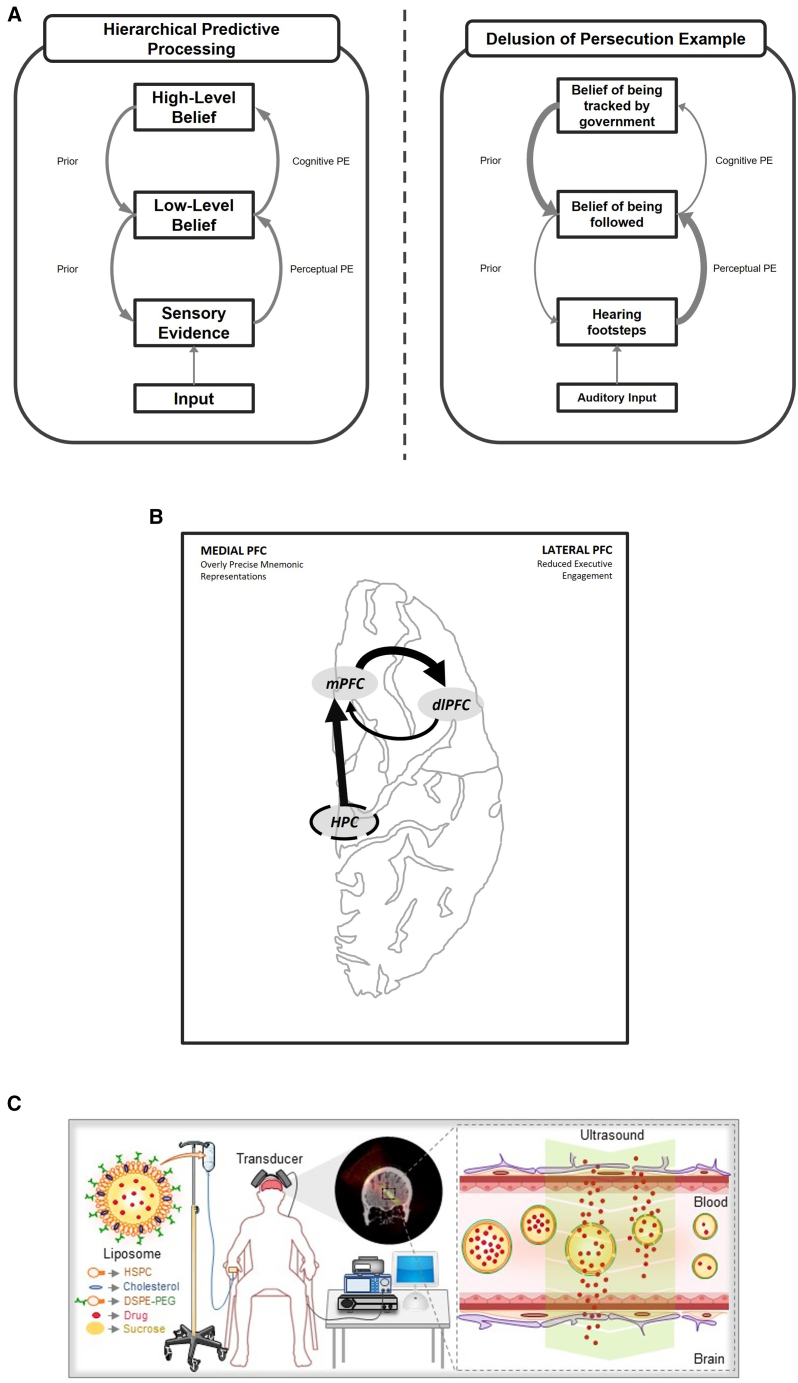
Hierarchical predictive processing framework and its relevance to delusions (A) Schematic model of hierarchical predictive processing in both a healthy context (left) and in the presence of a persecutory delusion (right). Arrows depict feedback signaling of priors and feedforward signaling of prediction errors (PEs), with arrow thickness indicating their relative precision. In the healthy model, prediction errors at both perceptual and cognitive levels guide continuous updating of beliefs in a balanced manner. By contrast, in the persecutory delusion example, auditory input, such as the sound of footsteps, leads to a low-level belief of being followed, which informs the high-level belief of being tracked by the government. Here, high-level priors gain excessive precision, compensating for the decreased precision priors of low-level beliefs and thus dominating interpretation of uncertain inputs. (B) Hypothesized medial lateral prefrontal cortex imbalance in schizophrenia. Schematic depiction of a network imbalance based on the hierarchical PPT algorithm. In this framework, the medial prefrontal cortex (mPFC) becomes dominated by memory-driven signals originating in the hippocampus (HPC), while the dorsolateral prefrontal cortex (dlPFC) shows diminished executive activity indicative of a mid-hierarchical deficit. As a result, evaluative processes in the medial area overshadow the lateral region’s executive functions, potentially giving rise to the delusional thinking observed in schizophrenia. (C) Ultrasound uncaging in treatment of psychiatric conditions. Identified network imbalances could potentially be targeted through ultrasonic drug uncaging: a patient visiting a clinical setting for usual treatment would receive an intravenous infusion of a liposomal ultrasound-sensitive nanocarrier encapsulating the drug of interest. Then, with an ultrasound transducer system placed on their scalp noninvasively targeting the region of interest, the drug would be released from the carriers as they circulate in the blood volume of the brain region of interest. The workflow is similar to that of transcranial magnetic stimulation (TMS), with the addition of the drug product infusion.

Given such parallels, one would expect PPT to have important insights into the neural mechanisms of schizophrenia. Indeed, a series of studies have formulated schizophrenia pathophysiology as resulting from imprecise predictive machinery (imprecise priors in Bayesian terms) combined with overly precise sensory data (high likelihoods).^70^^,^^88^^,^^89^^,^^90^^,^^91^ This results in a higher frequency of prediction errors, which the brain may interpret as hallucinatory experiences. While intriguing, this account introduces a paradox: delusional beliefs are highly resistant to change, suggesting increased rather than decreased weighting of priors. A recent proposal resolves this by extending classical PPT accounts to the cognitive domain through a hierarchical framework.^92^^,^^93^^,^^94^ Specifically, reduced precision is localized to the generative process of higher sensory areas but not to the associative areas above them. Instead, these areas may have increased precision, resulting in two types of prediction errors at different levels of hierarchy (Figure 4A). The increased perceptual prediction errors would therefore explain hallucinatory phenomena as in classical PPT, but the reduced cognitive prediction errors would explain the persistence of delusional thought content that cannot be explained by classical PPT. Therefore, hierarchical PPT aligns well with cognitive models of reasoning, mapping fast and slow thinking onto lower and higher hierarchical levels, and fits with known anatomical and physiological differences between associative and higher sensory cortical areas.^95^

A recent hierarchical PPT algorithmic model learned to model the process of schema formation and error-based learning.^96^ Despite its algorithmic nature, this model makes predictions regarding interactions between the medial (evaluative) and lateral (executive) divisions of the PFC. This is relevant to schizophrenia pathology; one possible instantiation of network imbalance could be between the medial and lateral PFC, with the medial regions driven by overly precise mnemonic representations, whereas lateral regions show reduced activity consistent with a mid-hierarchical deficit. This potential implementation (Figure 4B), which would focus on delusional thought processing, could simultaneously relate to hippocampal lesions resulting in delusions^97^ and the literature on impaired dorsolateral PFC function in schizophrenia.^98^ Thus, hierarchical PPT offers a testable way to link schizophrenia circuitry to symptoms and potential treatments.

### Computational models linking small circuits to networks to altered behavior in animal models relevant to schizophrenia

We described previously (in the “The role of formal computational theory in schizophrenia, linking cognitive theory to behavior” section) spatiotemporal DA dynamics across the striatum of the rodent and how it relates to agency and credit assignment.^64^ Such an intricate interplay between DA dynamics at multiple time scales and striatal subregions in the service of learning about agency could have profound implications for schizophrenia. Rather than considering how a single DA signal may be aberrant, such findings imply that it may be critical to control the timing and spatial specificity of DA terminals to assign credit to the correct neural circuit. Speculatively, disruptions in such signaling could give rise to inappropriate causal attribution to the agent’s own underlying corticostriatal circuits (e.g., an outcome that should be attributed to motor activity could be erroneously attributed to cognitive activity or vice versa). In rodents, these findings align with human studies linking DA signaling to learning values associated with agency^99^^,^^100^^,^^101^ and with neural network models implicating the need for multidimensional DA signaling for reinforcing appropriate striatal targets.^102^^,^^103^ Interestingly, findings across humans,^104^^,^^105^ non-human primates^106^ and rodents^107^ have shown dopaminergic locus of control within frontal thalamocortical networks, distinct from the classic corticostriatal system, augmenting the notion that DA impacts the brain at multiple levels including those responsible for the maintenance and switching of working memory.^25^^,^^50^^,^^107^ Notably, any blunt pharmacological DA manipulation would not restore the endogenous dynamics directed to particular striatal subregions needed for proper credit assignment.

Frontal thalamocortical systems have gained increasing attention in the pathophysiology of schizophrenia,^108^ while highlighting the value of animal models.^109^^,^^110^^,^^111^ These studies have amended the classical notion of the thalamus as a relay of peripheral inputs to the cortex, ushering in the notion of thalamic control of functional cortical connectivity.^112^ The impetus for this change is anchored in the unique structure of the thalamus, being composed of excitatory neurons that lack local recurrent connections.^113^^,^^114^^,^^115^^,^^116^^,^^117^ This feature results in long-range input-output connectivity patterns within the thalamus being the primary determinant of its computational properties.^118^^,^^119^^,^^120^^,^^121^^,^^122^ Specifically, while thalamic structures associated with early sensory processing (e.g., the lateral geniculate) receive strong driving inputs with little convergence, neurons in associative structures such as the mediodorsal thalamus receive convergent inputs from multiple prefrontal regions, enabling their integration as a bona fide thalamic computation. Such integration, whose properties have been modeled as a combination of input convergence and a unique Hebbian learning rule,^118^^,^^119^ results in the generation of “summary statistics,” including task context,^22^^,^^123^ input uncertainty,^107^ and context prediction errors.^123^ These signals are then projected back to prefrontal regions to control their ongoing dynamics. Of note, while thalamic neurons cannot excite each other directly, they are thought to inhibit one another via the thalamic reticular nucleus.^124^^,^^125^^,^^126^^,^^127^^,^^128^^,^^129^^,^^130^^,^^131^

Drawing from these systems neuroscience approaches,^132^ Huang et al. translated a mouse task designed to probe the role of mediodorsal thalamus in decision-making. The findings showed that mediodorsal neurons encoded cueing uncertainty based on convergent prefrontal inputs and drove prefrontal inhibition in a manner proportional to this summary statistic of signal reliability. Huang et al. found that subjects with schizophrenia showed a behavioral pattern qualitatively identical to optogenetic mediodorsal suppression in mice. What’s more, these behavioral deficits correlated with a more specific functional neuroimaging readout: the functional connectivity between the right mediodorsal thalamus and the right dorsolateral PFC. This identification of a neural readout of conflict-related executive dysfunction in schizophrenia was generalized to a larger dataset in a completely different cohort. The idea that a specific thalamic locus may be a target for intervention in schizophrenia is a tantalizing possibility. Next, we explore promising approaches for achieving this goal.

## New methods for studying and intervening at multiple levels

Although large advances in mapping circuits in preclinical models and advances in imaging methods in humans have and will continue to play an essential role in modeling across levels, direct interventions at these mapped levels in patients have been limited by a lack of interventional approaches. Over the last decade, there has been major progress in bridging this gap, through image-guided technologies that directly interact with brain circuit function including transcranial magnetic and direct or alternating current stimulation (TMS, TDC/TAC, or tcDCS or tcACS) and focused ultrasound.^133^ The transcranial electrical and magnetic stimulation techniques are widely used in clinical practice; however, they suffer from poor field penetration to the neural circuits of interest, given their relatively limited depth of penetration.^134^ In contrast, focused ultrasound can precisely reach nearly any brain region of interest, including medial prefrontal and striatal circuits.^135^ The following section describes progress in image-guided focused ultrasound and how its effectiveness may be improved through the parallel development of computational brain state models.

### Focused ultrasound as a method for circuit-specific drug targeting

To test hypotheses of how certain neural circuits and receptor systems contribute to schizophrenia, we need more precise controls over neural activity in target brain regions, with spatial, temporal, and receptor specificity. As noted previously, pharmacology provides the mainstay of current therapy for schizophrenia and yields powerful therapeutic effects in most patients, which can be adapted to specifically target a myriad of molecular receptors. However, side effects due to action of the drug at off-target regions are the norm for most pharmacologic treatments, limiting their therapeutic potential and complicating interpretation of how that drug affects the neural circuits and processes underlying schizophrenia for that particular patient.

Ideally, pharmacological treatments would retain their receptor-specific actions while achieving greater spatiotemporal precision, limiting drug exposure outside the target region to reduce side effects. An example of this approach is work by Airan et al. who have developed a technology for on-demand action of drugs at precise brain region of interest, at targeted time periods.^136^ They have created a suite of ultrasound-sensitive nanoparticulate drug carriers that can hold a drug bound until ultrasound induces release of the drug payload.^137^^,^^138^^,^^139^ With this system, they leverage focused ultrasound, which can target ultrasound energy to millimeter-scale regions of interest anywhere in the brain, with millisecond precision, to enable on-demand drug treatment at the right time and at the right brain target. In practice, the patient would receive an intravenous infusion of an ultrasound-sensitive drug-loaded nanocarrier while undergoing routine treatment in an ambulatory, intensive outpatient, or inpatient setting. Focused ultrasound would be applied under image guidance to the brain region(s) of interest including the key nodes of our computational framework. Importantly, this approach could be seamlessly integrated into in-person therapy session without disrupting the treatment workflow. Ultrasound would induce drug release from the nanocarrier as it traverses the blood volume of the region of interest. Then the freed drug would enter the brain, crossing the intact blood-brain barrier as it would otherwise normally do, but only at or near the sonicated site (Figure 4C). Airan et al. have validated this approach in animal models.^137^ They are now advancing toward a first-in-human application of this technology, targeting ketamine delivery to the anterior cingulate for chronic pain treatment.

### Integration of focused ultrasound and other circuit-level interventions with algorithmic models

Focused ultrasound used to directly modulate circuit function or target drug delivery can be a powerful method to validate algorithmic models and map their neural substrates. Let’s take the idea of paranoid delusions being the product of abnormal hierarchical PPT as suggested earlier. With focused ultrasound and its depth of precise focusing throughout the brain, we can selectively target different nodes of the neural hierarchy to test such hypotheses. By modulating excitatory-inhibitory balance in higher sensory or lower associative areas, it may be possible to enhance cognitive prediction errors sent to higher associative centers. Similarly, reducing activity in regions generating highly precise assumptions (e.g., hippocampus and medial PFC) could also prove beneficial. Crucially, these interventions should not rely solely on symptom reduction but should incorporate cognitive tasks that clarify when and why an approach fails. Therefore, having tasks that formalize slow and fast thinking and/or bias against disconfirmatory evidence would be a welcome approach.^140^

## The challenge of delivering new neuroscience innovation to the clinic

For regulatory approval and insurance coverage by third-party payers, interventions must demonstrate clear benefit in clinical trials. This is complicated by the large magnitude of contextual effects seen in most trials.^141^^,^^142^ If, as we posit, there are multiple circuits and pathways, at different scales, likely contributing to both the pathophysiology of disorder and mechanism of actions of treatments, we must also accept that contextual effects including the placebo effect and sequential effects of failed drug interventions can dramatically impact future treatment outcomes. We now know that several components of the placebo effect, expectations, conditioning, and therapeutic alliances, are mediated by, and themselves mediate, circuits involved with many of the core pathophysiological features of mental illness.^143^ Directly related to this, changes in belief updating and prediction error are thought to mediate part of placebo effect as well as play a central role in the pathophysiology of disorders like schizophrenia.^144^^,^^145^^,^^146^ It should therefore not be surprising to see large placebo effect sizes with clinical trial designs that focus on treatments targeting similar cognitive processes. When adequately masked, it could be very difficult to distinguish the effects of the drug or device from the behavioral or cognitive interventions alone.

A key challenge in demonstrating the efficacy of novel treatments is isolating the specific mechanisms driving clinical benefits. This issue is exemplified by 3,4-Methylenedioxymethamphetamine (MDMA)-assisted therapy for post-traumatic stress disorder (PTSD), where regulatory agencies struggle to determine whether the benefits stem from the drug’s plasticity-enhancing effects, the therapeutic alliance with the treaters, patient expectancy, or, we must be willing to consider, all or combinations of these factors.^147^^,^^148^ Asking such questions go beyond simply proving whether the treatment has superior efficacy over a placebo control. To combine therapeutics targeting various scalar levels of pathology, future clinical trialists must devise ways to disentangle these contributing factors, and regulatory bodies must develop new paradigms for paths to approval. It is also likely that the field will have to come together to better define the data needed to convince third-party payers. Furthermore, considerations must be given to ways of easily introducing these new modalities into existing healthcare delivery systems in a cost-effective manner. We have recently seen several examples of novel treatments such as IN esketamine for treatment-resistant depression that despite strong evidence of efficacy has limited use due to problems with patient access.^149^

## Conclusions and future outlook

Joan: Doctor, I can’t go back on the clozapine.

Doctor: Clozapine has helped you before, and it’s the best option we have for you right now. Can you share your concerns with me?

Joan: It’s too much for me. I do think it helps with voices, but I’m not me anymore.

Doctor: Can you expand on that Joan?

Joan: Sure—I totally see that it dulls the voices, and that relaxes me … somewhat. The issue is that I don’t feel the decisions I’m making are really mine anymore. Say I wanted to go out for a walk or go to the store to get ice cream. I normally know how to think about that choice, but not when I’m on clozapine. Do you see what I’m saying Doc?

Doctor: I understand what you’re saying, Joan, and think it is important we try to address this, but to be honest, we aren’t able to offer a clear description of how your decision-making may be affected with clozapine. We’re working hard on this and are making some progress on how decisions are affected in schizophrenia, but we have a long way to go. In the meantime, let’s work together on what medications we can use to give you some relief from the voices, but also keep you feeling like yourself and in control.

Contrasting the aforementioned conversation with that of Robert is meant to illustrate the heterogeneity within schizophrenia. There are individuals capable of eloquently describing what they experience, yet research lacks the tools and frameworks to fully capture these nuances. This underscores the need for integrated models that account for the full breadth of lived experiences in ways that facilitate individualized interventions and mitigate issues of “illusory generalizability” where group-based findings fail to reflect individual experiences. By linking computational, neural, and behavioral data, we can develop models that allow for more accurate predictions, applicable to unique manifestations of disease states at the individual level.^150^ Moreover, computational models offer the potential to bridge symptomatology and underlying neural mechanisms, providing a clearer understanding of how specific brain circuit disruptions translate into observable behaviors and symptoms. As we refine these models, they can assist in guiding the selection of personalized interventions—whether pharmacological, neuromodulatory, or behavioral—by identifying strategies that would be effective to individual’s unique cognitive and neural profile. This personalized approach would permit real-time treatment plan adjustments, resulting in better management of schizophrenia’s evolving course.

In this piece, we have provided an integrative perspective that centers algorithmic models of behavior as a critical node between brain science and mental health, using schizophrenia as a case example. This node provides the necessary language to translate symptoms into computational units and portray them in a manner that can be independently targeted with different treatment modalities. While astute clinicians already navigate psychiatric complexities in a similar way, this approach offers a structured framework that accommodates emerging advances in neural measurements and interventions.

We envision a future where integrative, algorithm-driven models revolutionize mental health care, particularly in treating complex conditions such as schizophrenia. The next 20 years hold immense potential for advances in treatment, but realizing this potential requires a commitment to implementing frameworks that capture the wide spectrum of patient experiences. Only through this concerted effort from the larger clinical and research community will we be able to address the needs of individuals like Robert and Joan in profound and transformative ways, ultimately delivering the life-changing solutions that they, and countless others, deserve.

## Acknowledgments

We thank the James S. McDonnel Foundation for hosting the workshop, which inspired the ideas outlined in this perspective. The patient stories depict “composite patients” that are based on real patient encounters.

## Declaration of interests

D.O. has served on advisory board for Rapport Therapeutics.
